# Supplementary material for: Fused Triazinobenzimidazoles Bearing Heterocyclic Moiety: Synthesis, Structure Investigations, and In Silico and In Vitro Biological Activity
Source: Molecules. 2023 Jun 27;28(13):5034. doi: 10.3390/molecules28135034 (PMC10343767; doi:10.3390/molecules28135034)
Supplement: Supplementary file 1 [file molecules-28-05034-s001.zip › molecules-2455978-supplementary.pdf]

**Fused Triazinobenzimidazoles Bearing Heterocyclic Moiety: Synthesis, Structure Investigations, In silico and In vitro Biological activity**

**Kameliya Anichina[1*], Nikolai Georgiev[1*], Nikolay Lumov[2], Dimitar Vuchev[3], Galya Popova-Daskalova[3], Georgi Momekov[4], Emiliya Cherneva[2,4], Rositsa Mihaylova[4], Anelia Mavrova[1], Stela Atanasova-Vladimirova[5], Iskra Piroeva [5], Denitsa Yancheva[2]**

[1] University of Chemical Technology and Metallurgy, Department of Organic Synthesis, 8 Kliment Ohridski Blvd., 1756 Sofia, Bulgaria
[2] Institute of Organic Chemistry with Centre of Phytochemistry, Bulgarian Academy of Sciences, Acad. G. Bonchev str. Bl. 9, 1113 Sofia, Bulgaria
[3] Department of Infectious Diseases, Parasitology and Tropical Medicine, Medical University,15A Vasil Aprilov Blvd., 4002 Plovdiv, Bulgaria
[4] Faculty of Pharmacy, Medical University of Sofia, 2 Dunav Str., 1000, Sofia, Bulgaria
[5] Institute of Physical Chemistry, Bulgarian Academy of Sciences, build. 11, 1113, Sofia, Bulgaria

* nikigeorgiev@uctm.edu (N.G.);
* kameliya_anichina@uctm.edu (K.A.);

**Contents**

**Figure S1.** Molecular structure of conformers and tautomeric forms of compounds **3a-f** optimized at DFT (B3LYP/6-311++G**) level of theory, in water medium, and the corresponding total ($G_{tot}$) and relative ($\Delta G$) free energies………………………..……….......................................................................................................page 2

**Table S1**. Chemical shifts for the three possible tautomer forms of compound **3b** predicted by GIAO method using B3LYP functional and 6-311++G** basis set calculations in DMSO solvent and experimental data for **3b**……………………………………………………………………………………………….. page 5

**Table S2.** Molecular properties of the tested compound calculated by using the Molinspiration tool: partition coefficients (milogP), molecular weight (MW) [g/mol], topologic polar surface area (TPSA) [Å$^2$], molecular volume (Vol.) [Å$^3$], sum of OH and NH H-bond donors (NHD) and sum of O and N H-bond acceptors (NHA)………………………………………………………………………………………page 6

**Figures. S2–S28**. IR, NMR and HRMS (ESI) spectra of the synthesized compounds ……………………………………………………………………………………………………pages 7-24

**Figure S1.** Molecular structure of conformers and tautomeric forms of compounds **3a-f** optimized at DFT (B3LYP/6-311++G**) level of theory, in water medium, and the corresponding total ($G_{tot}$) and relative ($\Delta G$) free energies

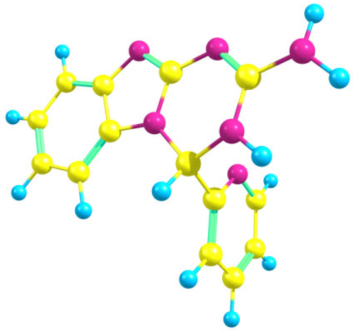
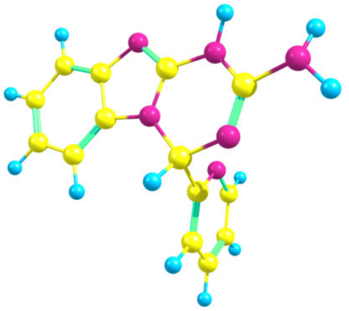
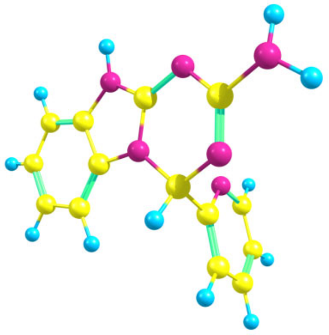

**3a-A** **3a-B** **3a-C**

| Compound | G (a.u.) | ΔG (a.u.) | ΔG (kJ.mol$^{-1}$) |
|---|---|---|---|
| **3a-A** | -869.288898 | 0 | 0 |
| **3a-B** | -869.284540 | 0.004358 | 11.441929 |
| **3a-C** | -869.283776 | 0.005122 | 13.447811 |

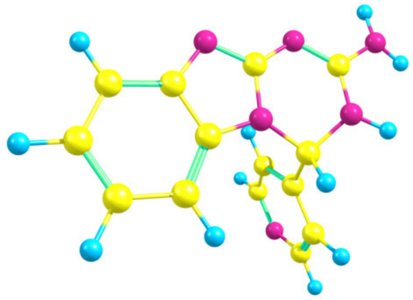
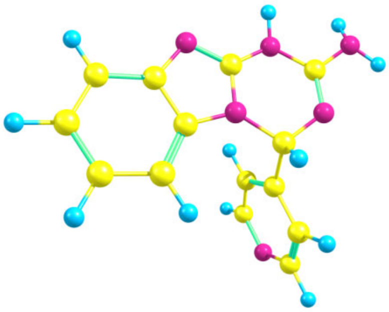
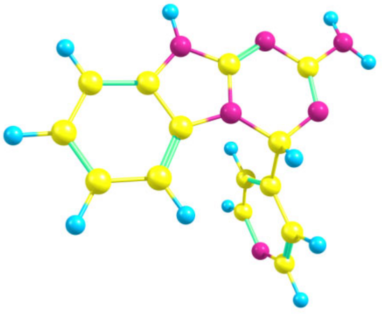

**3b-A** **3b-B** **3b-C**

| Compound | G (a.u.) | ΔG (a.u.) | ΔG (kJ.mol$^{-1}$) |
|---|---|---|---|
| **3b-A** | -869.285579 | 0 | 0 |
| **3b-B** | -869.282275 | 0.003304 | 8.674652 |
| **3b-C** | -869.281724 | 0.003855 | 10.121303 |

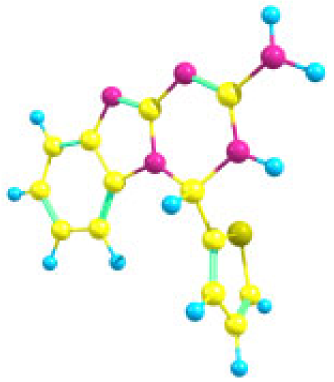

**3c-A**

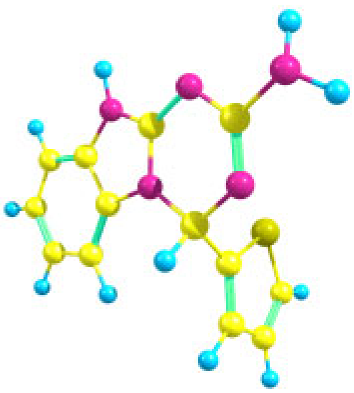

**3c-B**

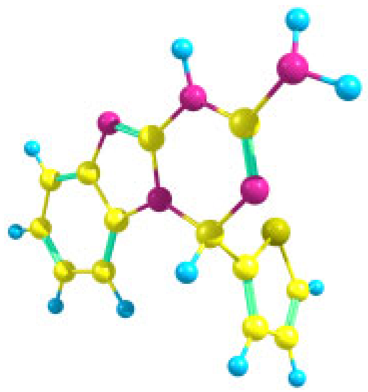

**3c-C**

| Compound | G (a.u.) | ΔG (a.u.) | ΔG (kJ.mol$^{-1}$) |
|---|---|---|---|
| **3c-A** | -1174.027913 | 0 | 0 |
| **3c-C** | -1174.023261 | 0.004652 | 12.213826 |
| **3c-B** | -1174.022969 | 0.004944 | 12.980472 |

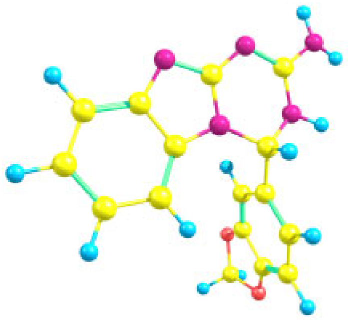

**3d-A**

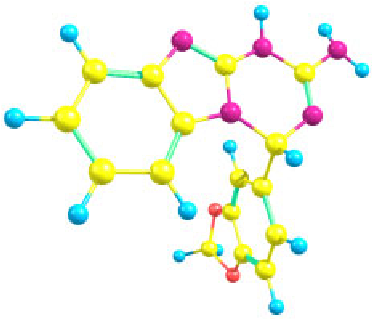

**3d-B**

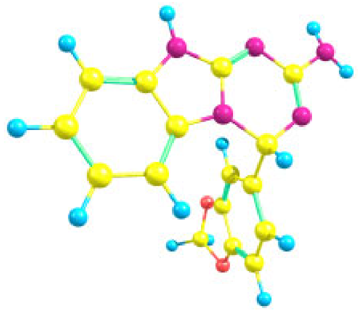

**3d-C**

| Name | G | ΔG (a.u.) | ΔG (kJ.mol$^{-1}$) |
|---|---|---|---|
| **3d-A** | -1041.804493 | 0 | 0 |
| **3d-C** | -1041.799275 | 0.005218 | 13.699859 |
| **3d-B** | -1041.798847 | 0.005646 | 14.823573 |

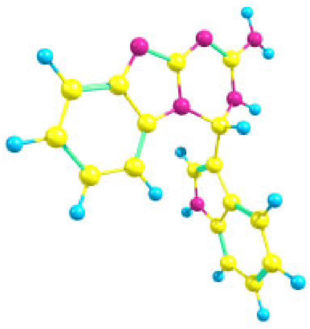

**3e-A**

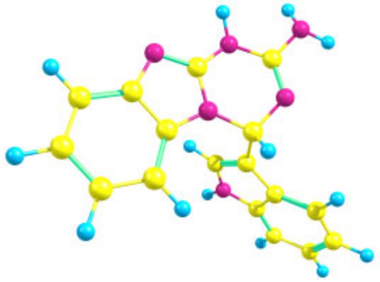

**3e-B**

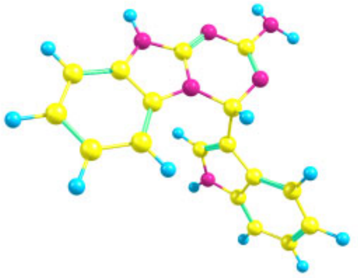

**3e-C**

| Compound | G (a.u.) | ΔG(a.u.) | ΔG (kJ.mol$^{-1}$) |
|---|---|---|---|
| **3e-A** | -984.813628 | 0 | 0 |
| **3e-B** | -984.807932 | 0.005696 | 14.964848 |
| **3e-C** | -984.807792 | 0.005836 | 15.322418 |

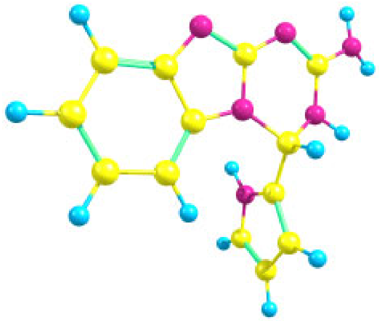

**3f-A**

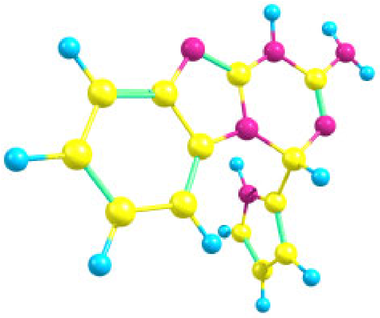

**3f-C**

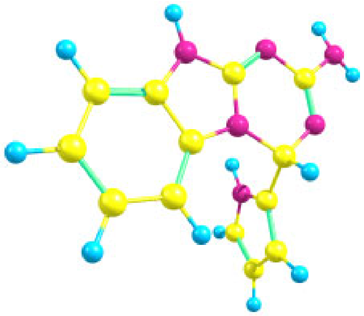

**3f-B**

| Compound | G (a.u.) | ΔG(a.u.) | ΔG (kJ.mol$^{-1}$) |
|---|---|---|---|
| **3f-A** | -831.173767 | 0 | 0 |
| **3f-C** | -831.168684 | 0.005083 | 13.345417 |
| **3f-B** | -831.167981 | 0.005786 | 15.191143 |

**Table S1**. Chemical shifts for the three possible tautomer forms of compound **3b** predicted by GIAO method using B3LYP functional and 6-311++G** basis set calculations in DMSO solvent and experimental data for **3b.**

| **C Atom №** | **$\delta_{Exp.}$ in DMSO-$d_6$** | **Tautomeric form A** | | **Tautomeric form B** | | **Tautomeric form C** | |
|---|---|---|---|---|---|---|---|
| | | B3LYP/6-311++G** DMSO | **chem. shift** | B3LYP/6-311++G** DMSO | **chem. shift** | B3LYP/6-311++G** DMSO | **chem. shift** |
| **C-8** | 119.8 | 56.1324 | 128.5 | 55.2219 | 129.4 | 54.5867 | 130.0 |
| **C-7** | 121.3 | 57.7005 | 126.9 | 56.821 | 127.8 | 55.3394 | 129.2 |
| **C-6** | 108.5 | 71.4223 | 113.2 | 69.3717 | 115.2 | 69.5722 | 115.0 |
| **C-11** | 121.7 | 45.3291 | 139.3 | 45.5356 | 139.0 | 47.9614 | 136.6 |
| **C-12** | 131.4 | 31.7525 | 152.8 | 33.9103 | 150.7 | 47.9923 | 136.6 |
| **C-9** | 149.0 | 31.014 | 153.6 | 60.5919 | 124.0 | 68.4769 | 116.1 |
| **C-2** | 155.7 | 24.0094 | 160.6 | 31.063 | 153.5 | 24.8183 | 159.8 |
| **C-4** | 64.7 | 111.4864 | 73.1 | 105.537 | 79.0 | 103.9146 | 80.7 |
| **C-13** | 150.9 | 25.2939 | 159.3 | 34.094 | 150.5 | 21.7549 | 162.8 |
| **H Atom №** | | | | | | | |
| **H-C8** | 6.85 | 24.5595 | 7.4 | 24.4273 | | 24.5214 | |
| **H-C7** | 6.84 | 24.665 | 7.3 | 24.6377 | 7.3 | 24.7259 | 7.2 |
| **H-C6** | 6.97 | 24.8803 | 7.1 | 25.0096 | 7.0 | 25.3012 | 6.7 |
| **H-C9** | 7.26 | 24.2299 | 7.7 | 24.2244 | 7.7 | 24.5795 | 7.4 |
| **H-C4** | 6.91 | 25.2514 | 6.7 | 25.1151 | 6.9 | 25.2223 | 6.7 |

**Table S2.** Molecular properties of the tested compound calculated by using the Molinspiration tool: partition coefficients (milogP), molecular weight (MW) [g/mol], topologic polar surface area (TPSA) [Å$^2$], molecular volume (Vol.) [Å$^3$], sum of OH and NH H-bond donors (NHD) and sum of O and N H-bond acceptors (NHA)

| Name | miLogP | TPSA | natoms | MW | nOH | nOHNH | nviolations | nrotb | vol |
|---|---|---|---|---|---|---|---|---|---|
| **3a-A** | 1.26 | 81.13 | 20 | 264.29 | 6 | 3 | 0 | 1 | 229.44 |
| **3a-B** | 1.26 | 81.13 | 20 | 264.29 | 6 | 3 | 0 | 1 | 229.44 |
| **3a-C** | 1.43 | 84.37 | 20 | 264.29 | 6 | 3 | 0 | 1 | 229.44 |
| **3b-A** | 1.14 | 81.13 | 20 | 264.29 | 6 | 3 | 0 | 1 | 229.44 |
| **3b-B** | 1.14 | 81.13 | 20 | 264.29 | 6 | 3 | 0 | 1 | 229.44 |
| **3b-C** | 1.31 | 84.37 | 20 | 264.29 | 6 | 3 | 0 | 1 | 229.44 |
| **3c-A** | 2.33 | 68.24 | 19 | 269.33 | 5 | 3 | 0 | 1 | 224.3 |
| **3c-B** | 2.33 | 68.24 | 19 | 269.33 | 5 | 3 | 0 | 1 | 224.3 |
| **3c-C** | 2.50 | 71.48 | 19 | 269.33 | 5 | 3 | 0 | 1 | 224.3 |
| **3d-A** | 2.32 | 86.71 | 23 | 307.31 | 7 | 3 | 0 | 1 | 257.52 |
| **3d-B** | 2.32 | 86.71 | 23 | 307.31 | 7 | 3 | 0 | 1 | 257.52 |
| **3d-C** | 2.49 | 89.94 | 23 | 307.31 | 7 | 3 | 0 | 1 | 257.52 |
| **3e-A** | 2.58 | 84.03 | 23 | 302.34 | 6 | 4 | 0 | 1 | 262.57 |
| **3e-B** | 2.58 | 84.03 | 23 | 302.34 | 6 | 4 | 0 | 1 | 262.57 |
| **3e-C** | 2.75 | 87.27 | 23 | 302.34 | 6 | 4 | 0 | 1 | 262.57 |
| **3f-A** | 1.58 | 84.03 | 19 | 252.28 | 6 | 4 | 0 | 1 | 218.58 |
| **3f-B** | 1.58 | 84.03 | 19 | 252.28 | 6 | 4 | 0 | 1 | 218.58 |
| **3f-C** | 1.76 | 87.27 | 19 | 252.28 | 6 | 4 | 0 | 1 | 218.58 |

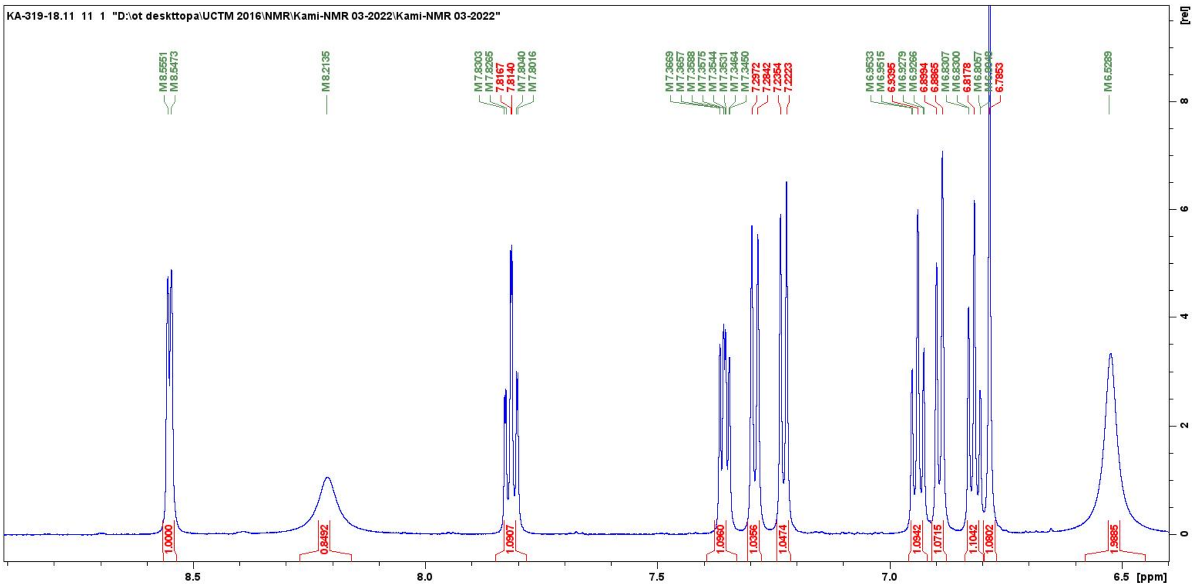

**Figure S2.** $^{1}H$ NMR spectrum of compound **3a**

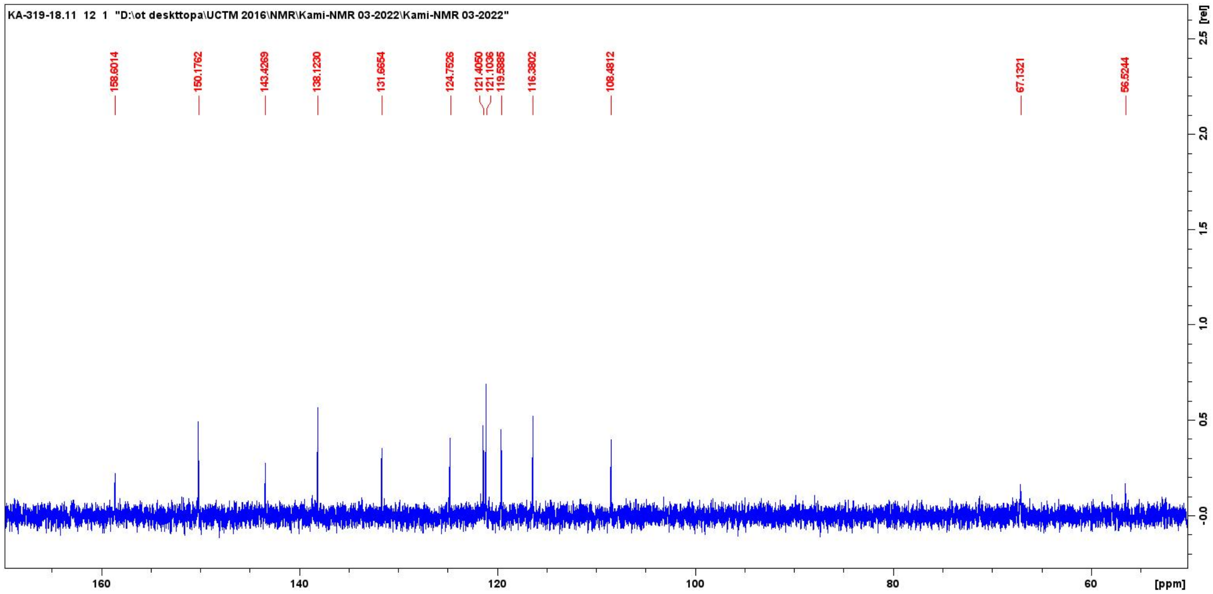

**Figure S3.** $^{13}C$ NMR spectrum of compound **3a.**

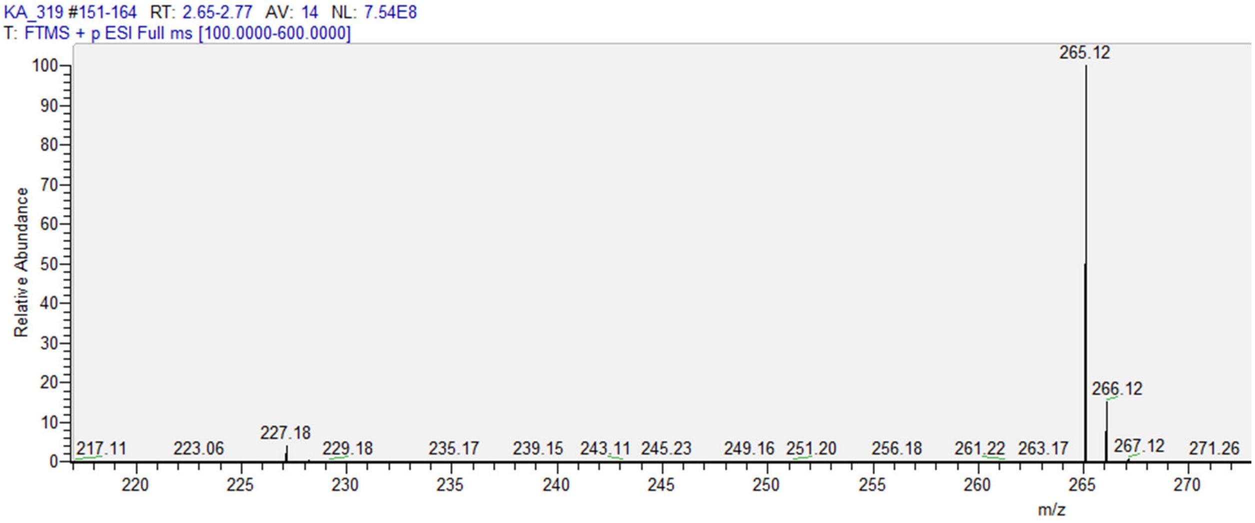


**Figure S4.** HRMS (ESI) spectrum of compound **3a**.

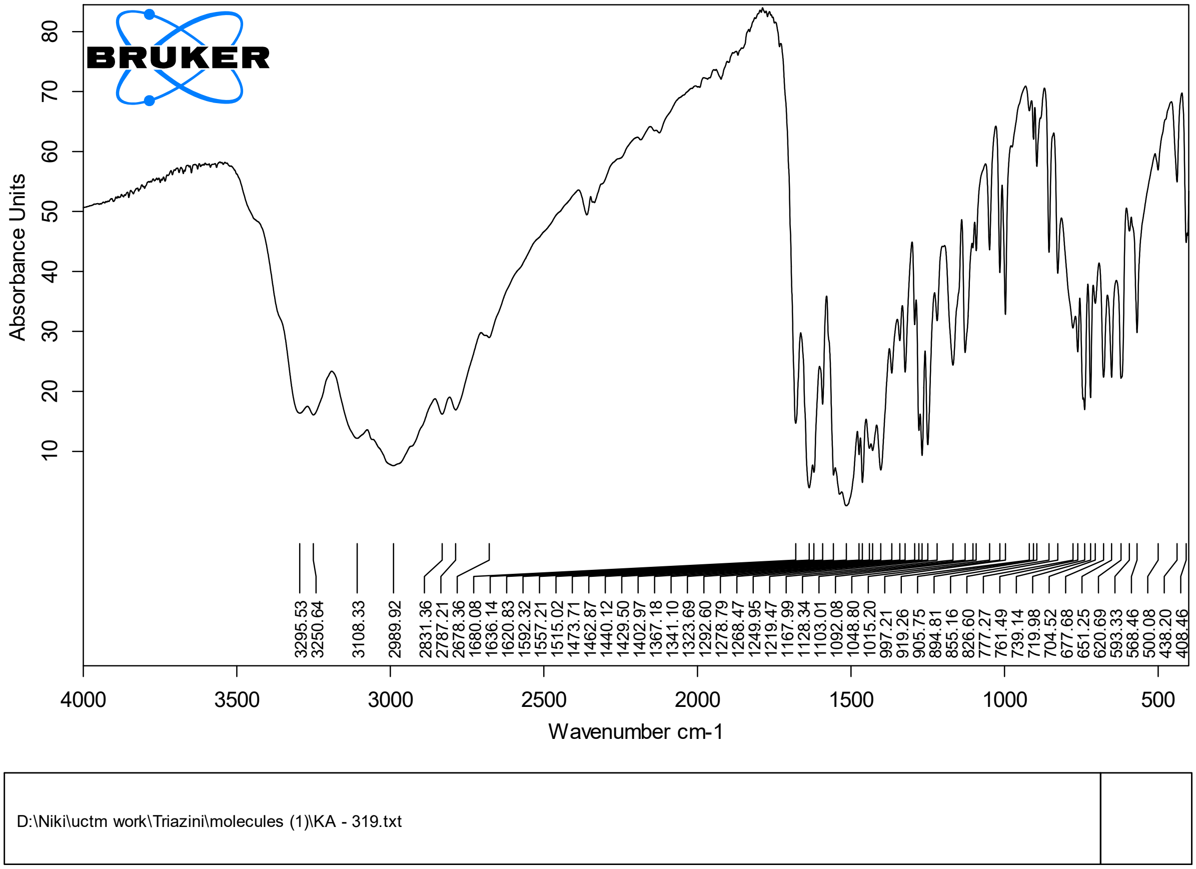


**Figure S5a.** IR spectrum of compound **3a.**

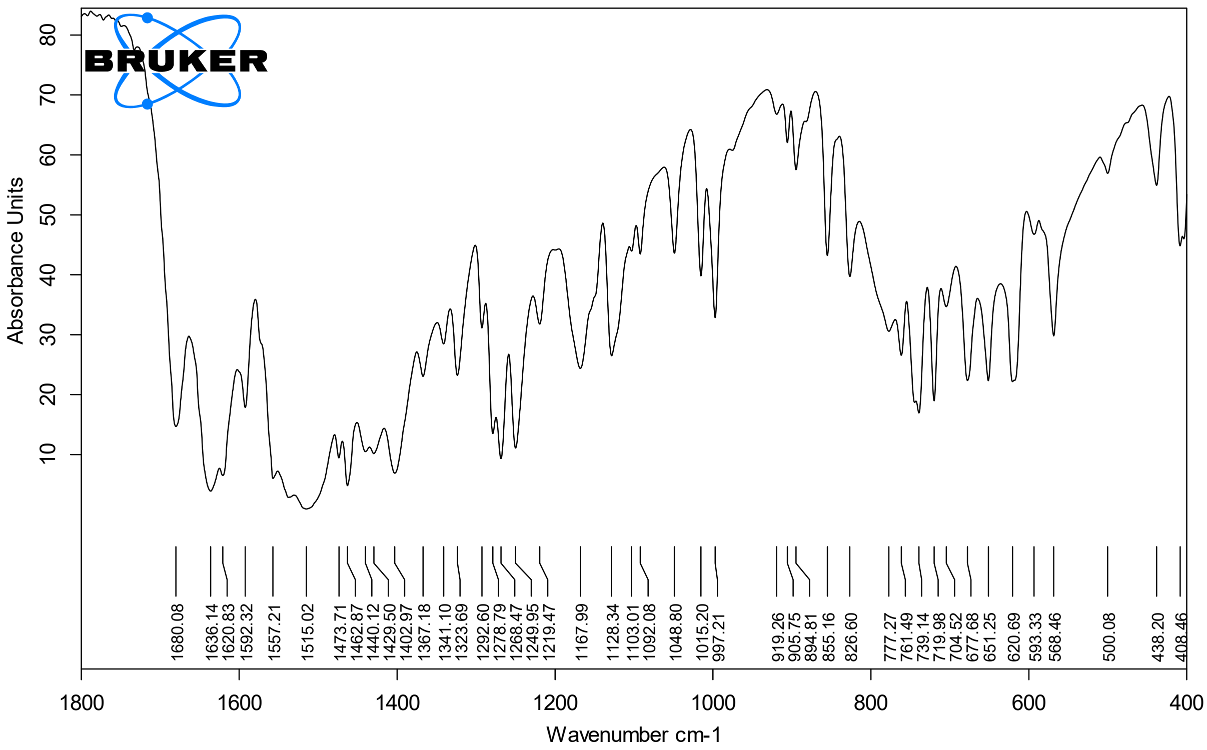


D:\Niki\uctm work\Triazini\molecules (1)\KA - 319.txt

**Figure S5b.** IR spectrum of compound **3a.**

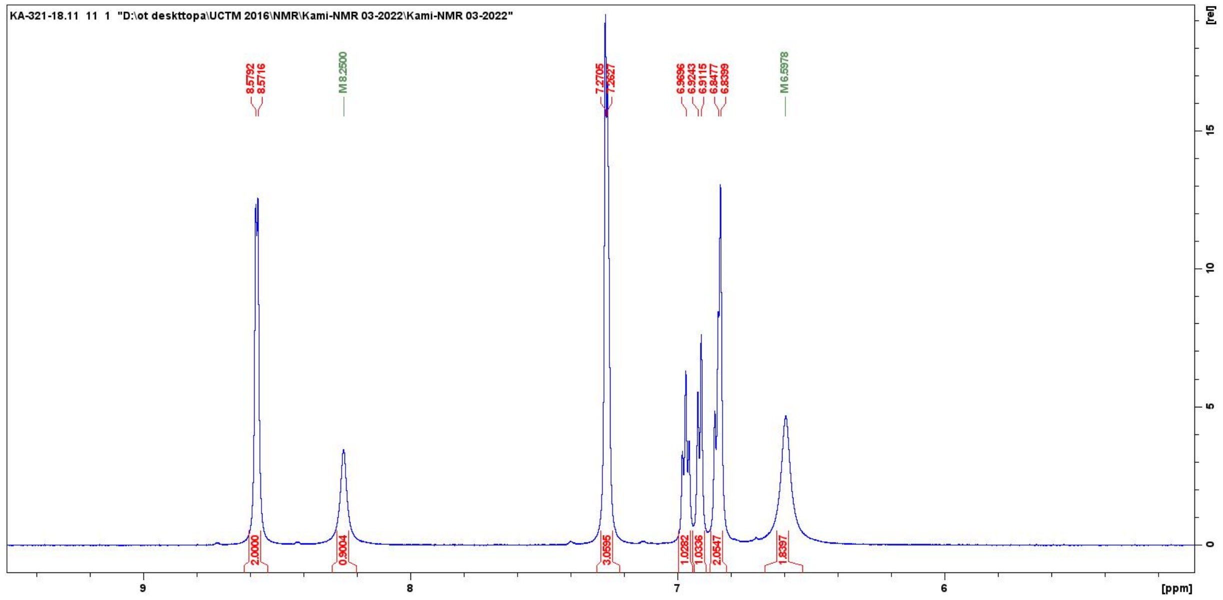


**Figure S6.** $^{1}$H NMR spectrum of compound **3b.**

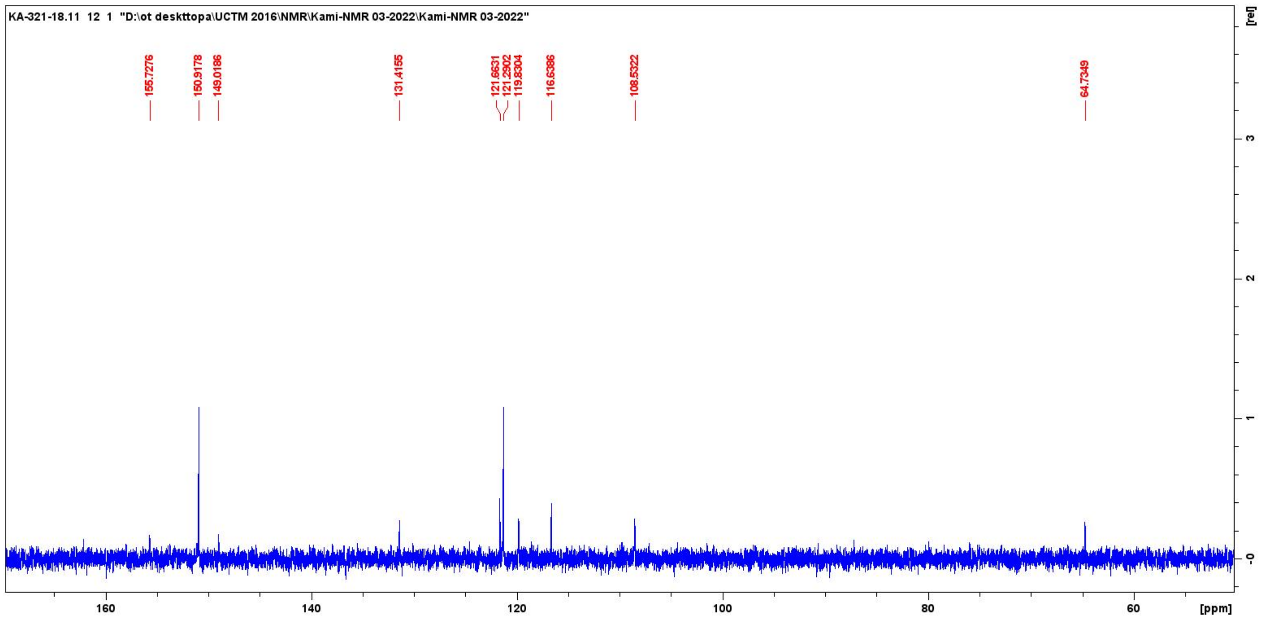


**Figure S7.** $^{13}$C NMR spectrum of compound **3b.**

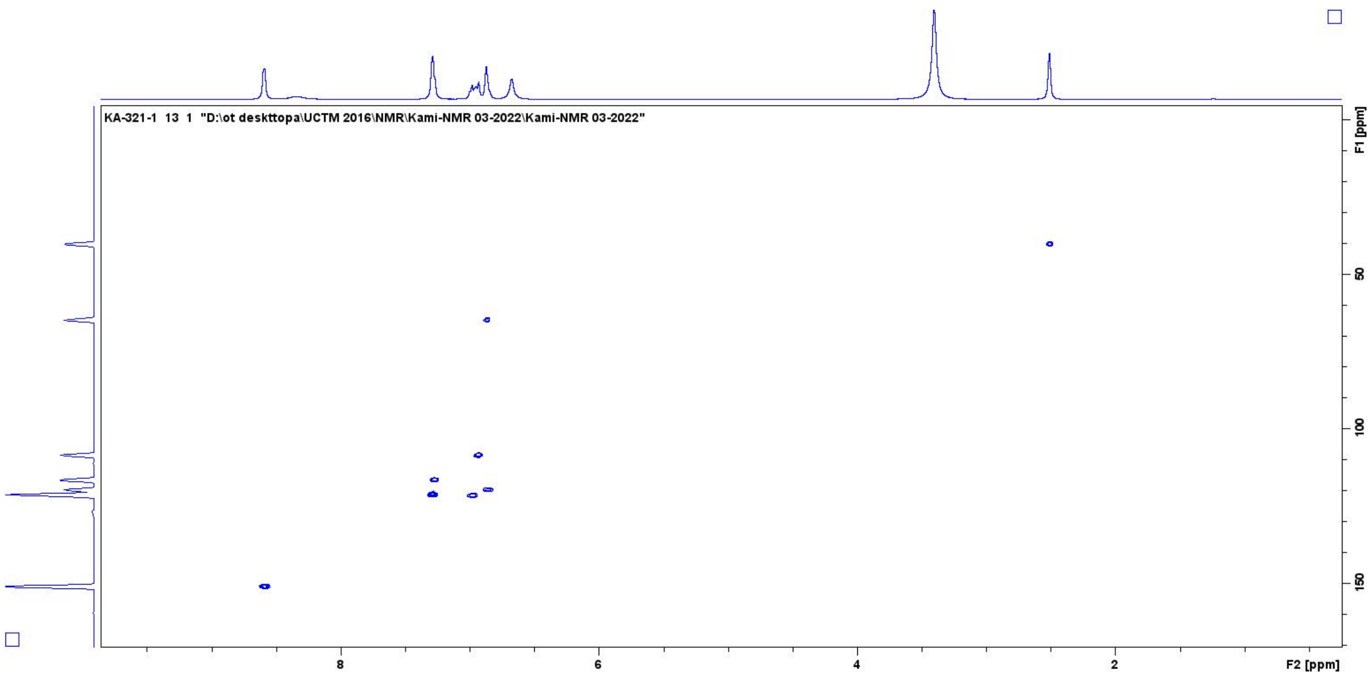


**Figure S8.** HSQC spectrum of compound **3b**

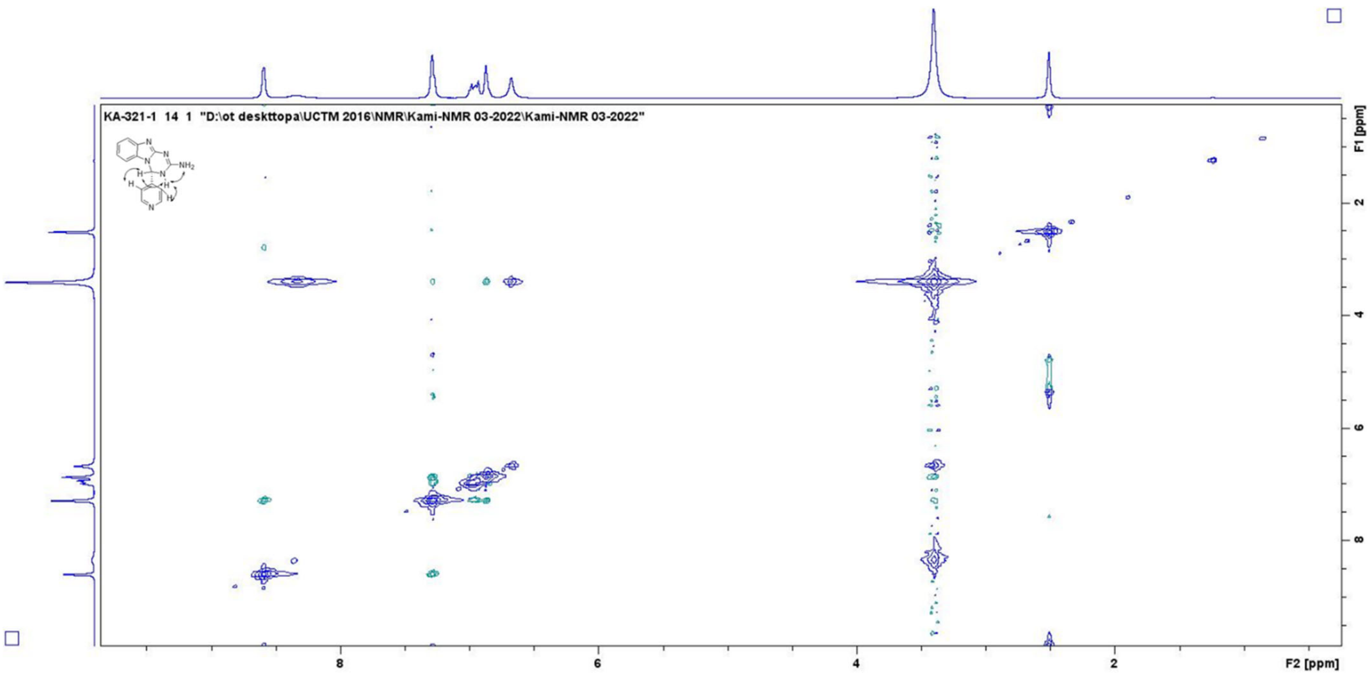


**Figure S9.** NOESY spectrum of compound **3b.**

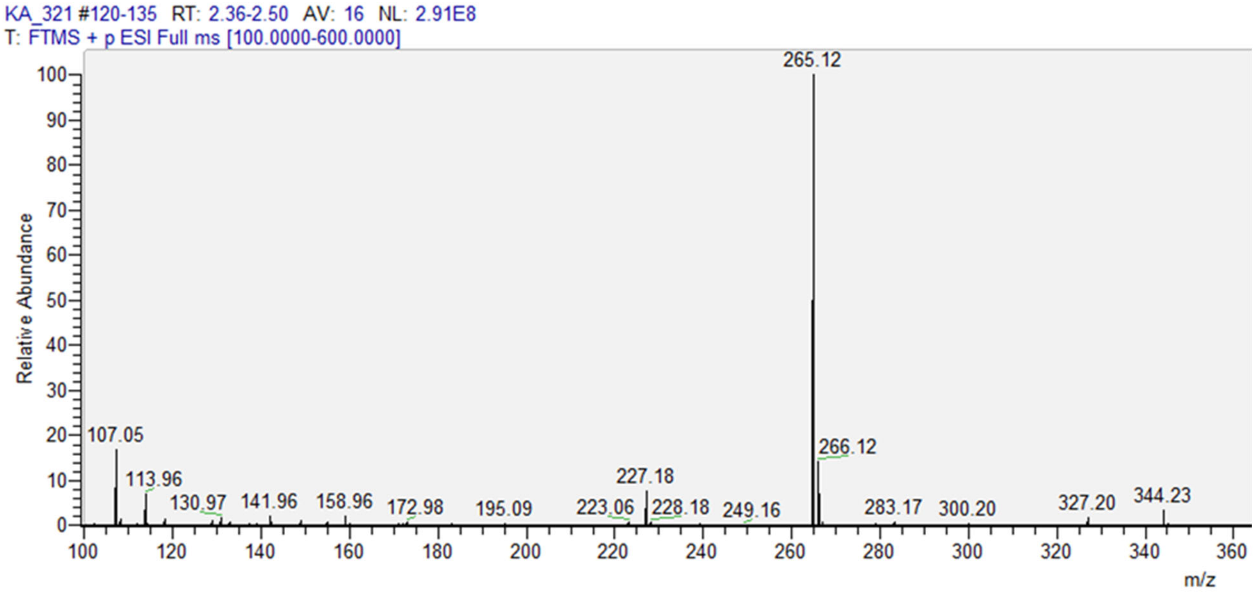


**Figure S10.** HRMS (ESI) spectrum of compound **3b**.

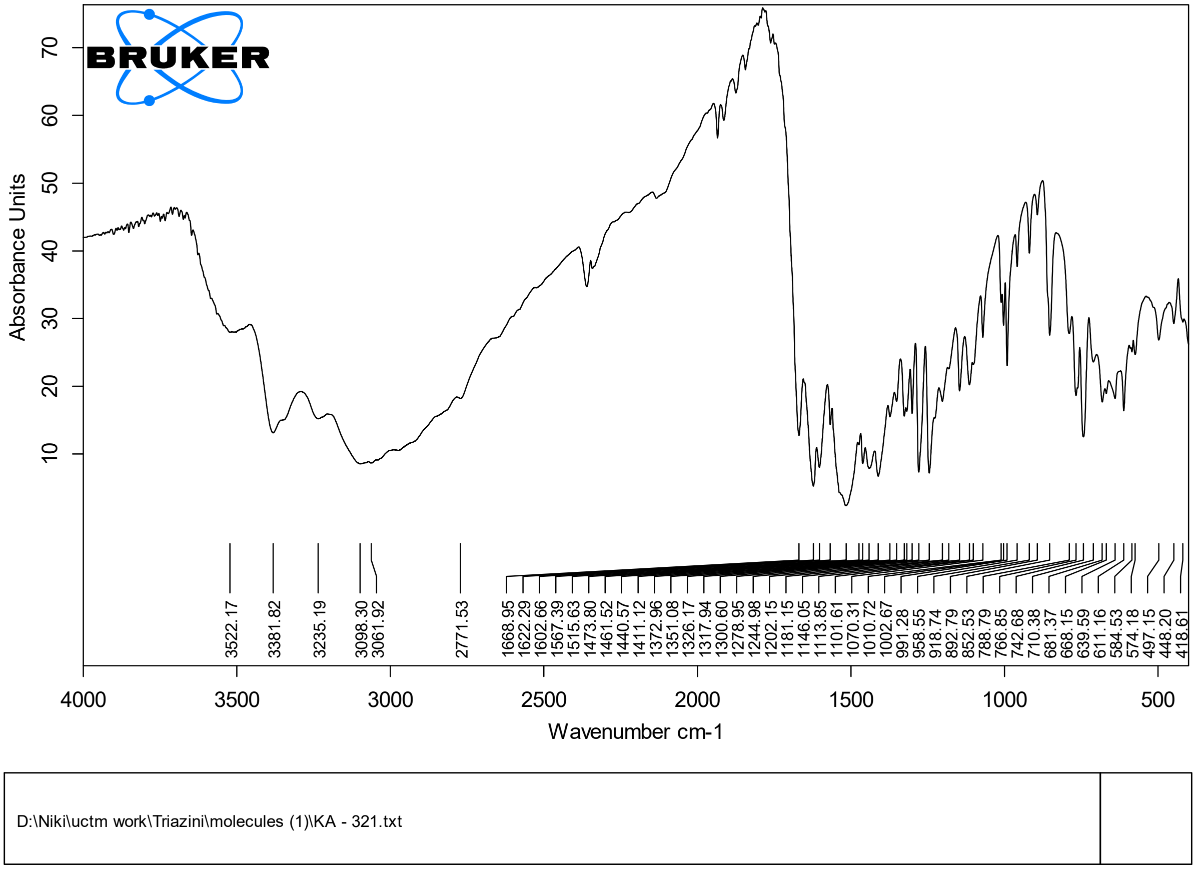


**Figure S11a.** IR spectrum of compound **3b.**

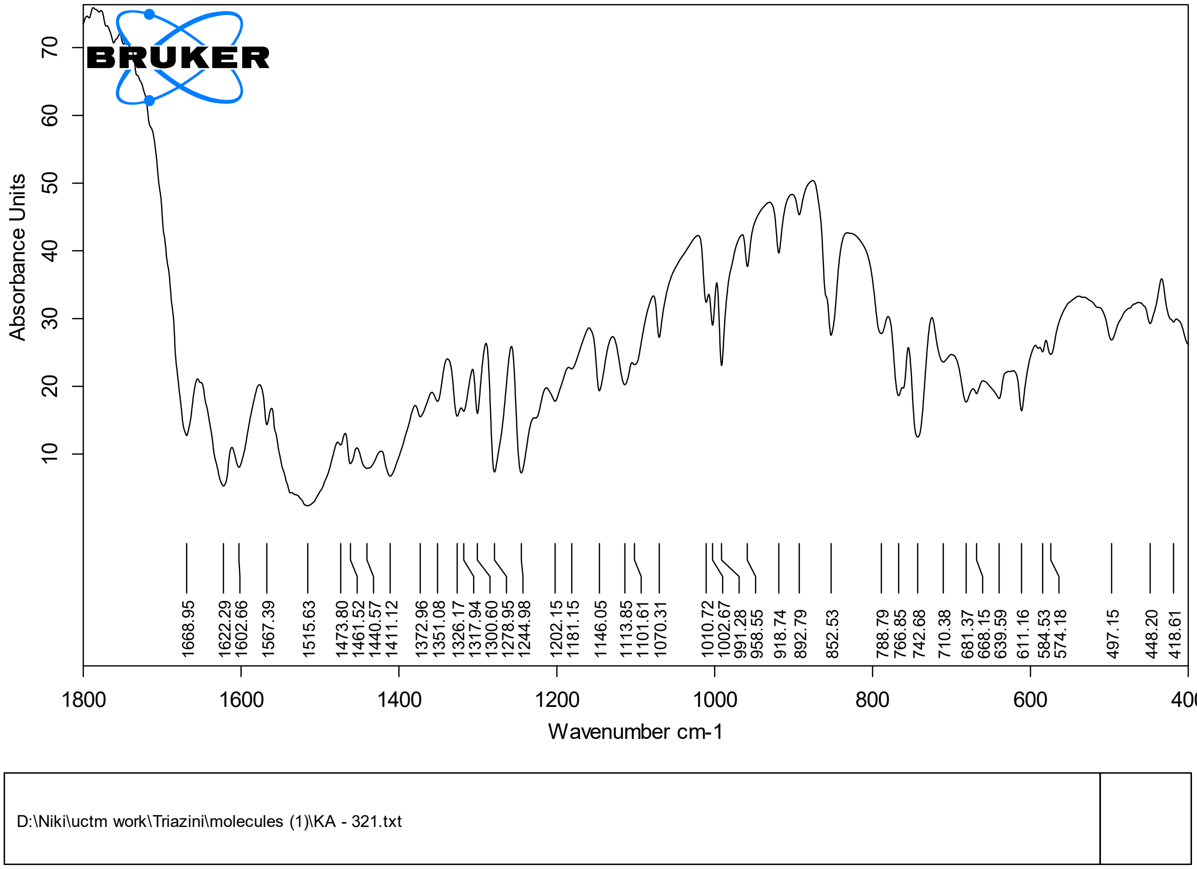


**Figure S11b.** IR spectrum of compound **3b.**

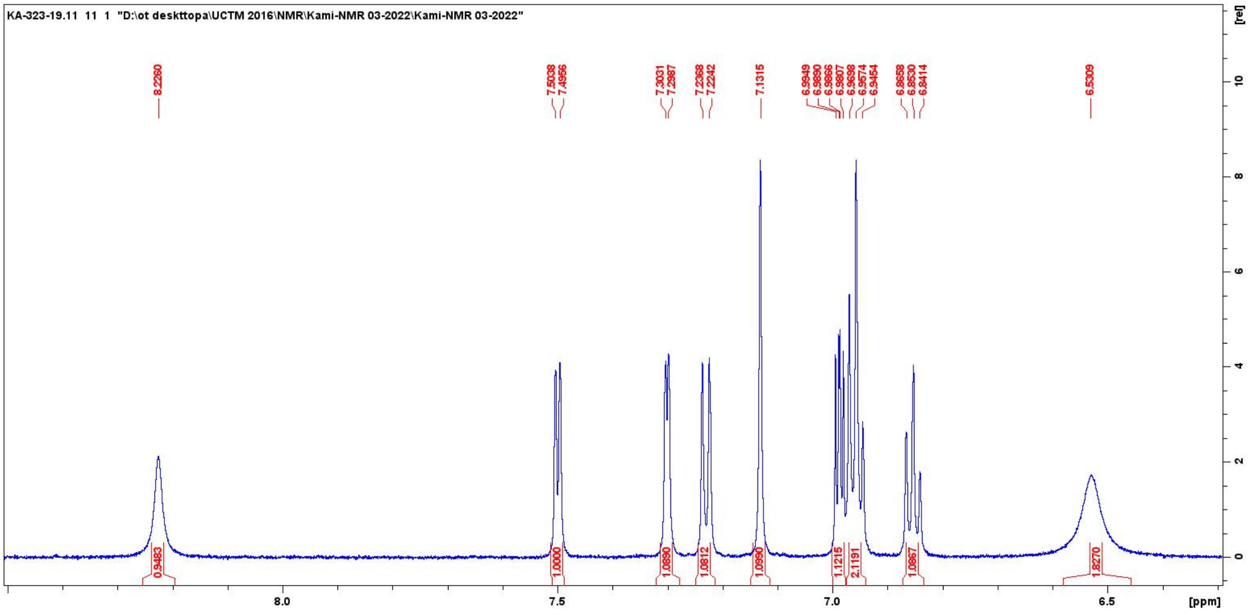


**Figure S12.** $^{1}$H NMR spectrum of compound **3c**

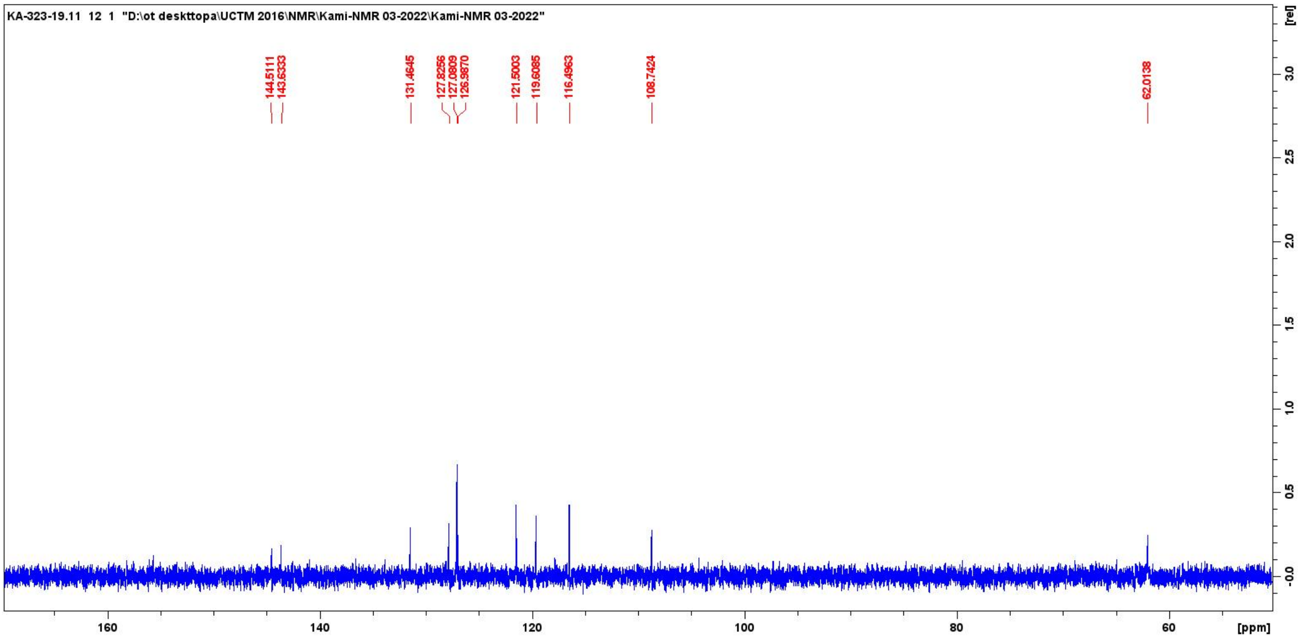


**Figure S13.** $^{13}$C NMR spectrum of compound **3c**

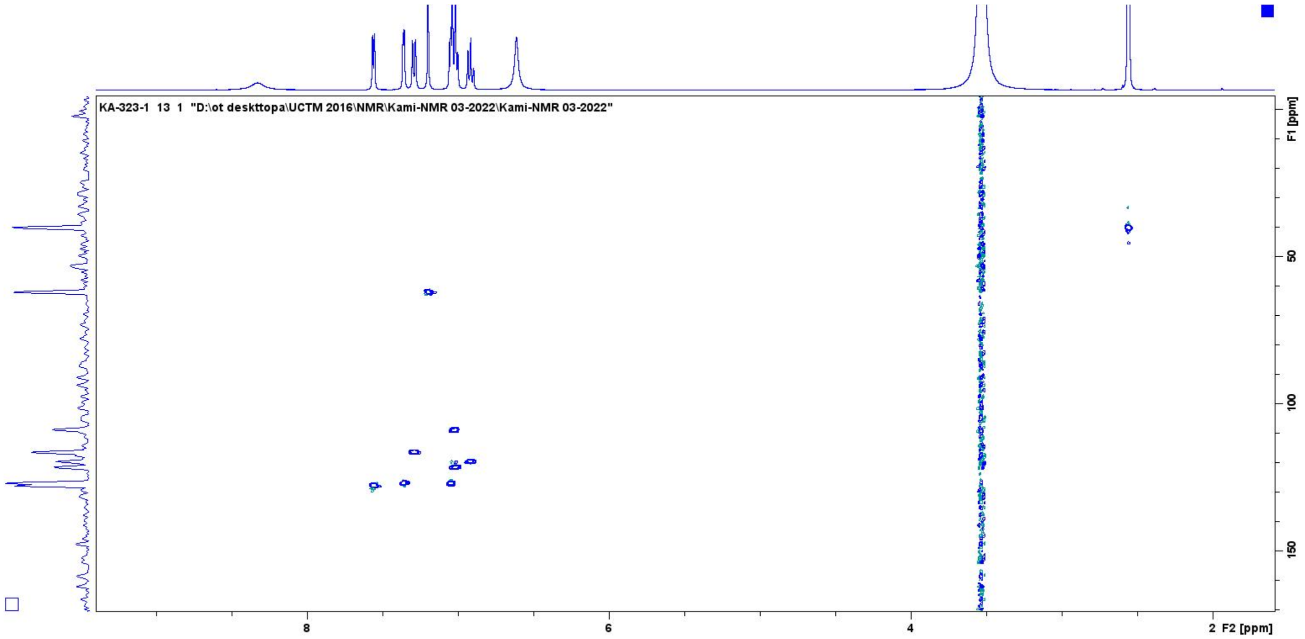


**Figure S14.** HSQC spectrum of compound **3c.**

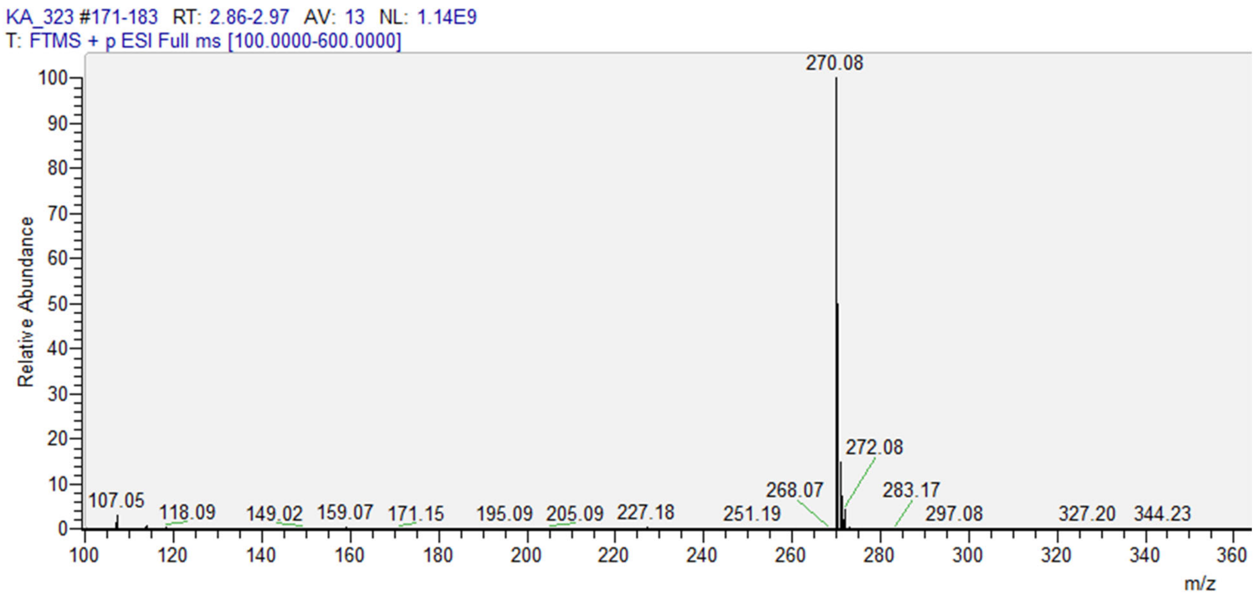


**Figure S15.** HRMS (ESI) spectrum of compound **3c.**

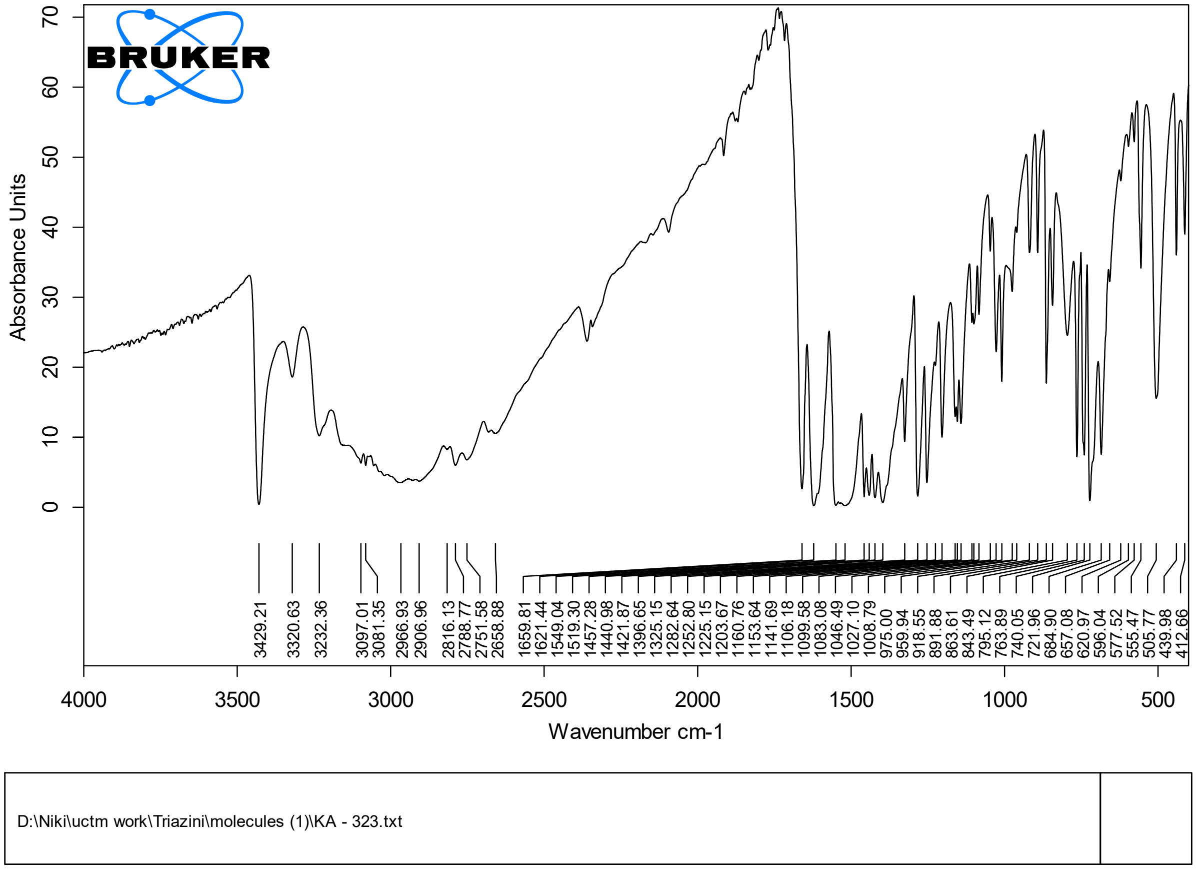


**Figure S16a.** IR spectrum of compound **3c.**

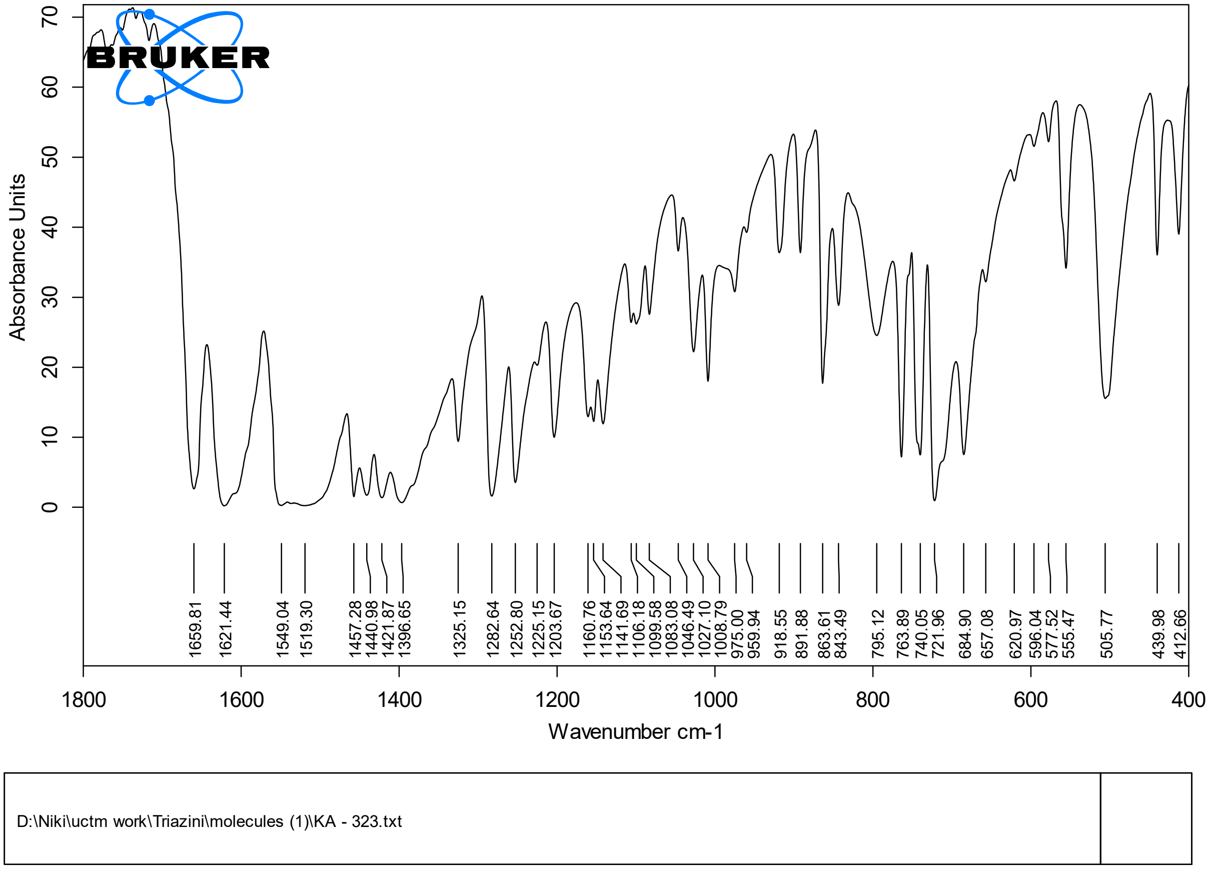


**Figure S16b.** IR spectrum of compound **3c.**

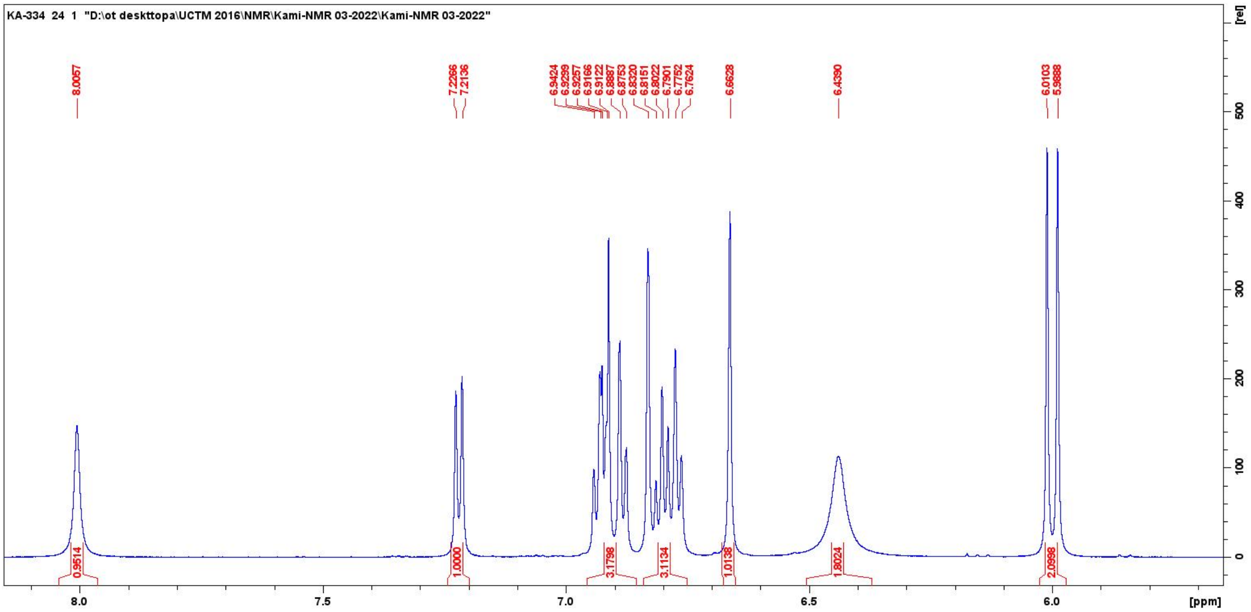


**Figure S17.** $^{1}H$ NMR spectrum of compound **3d**

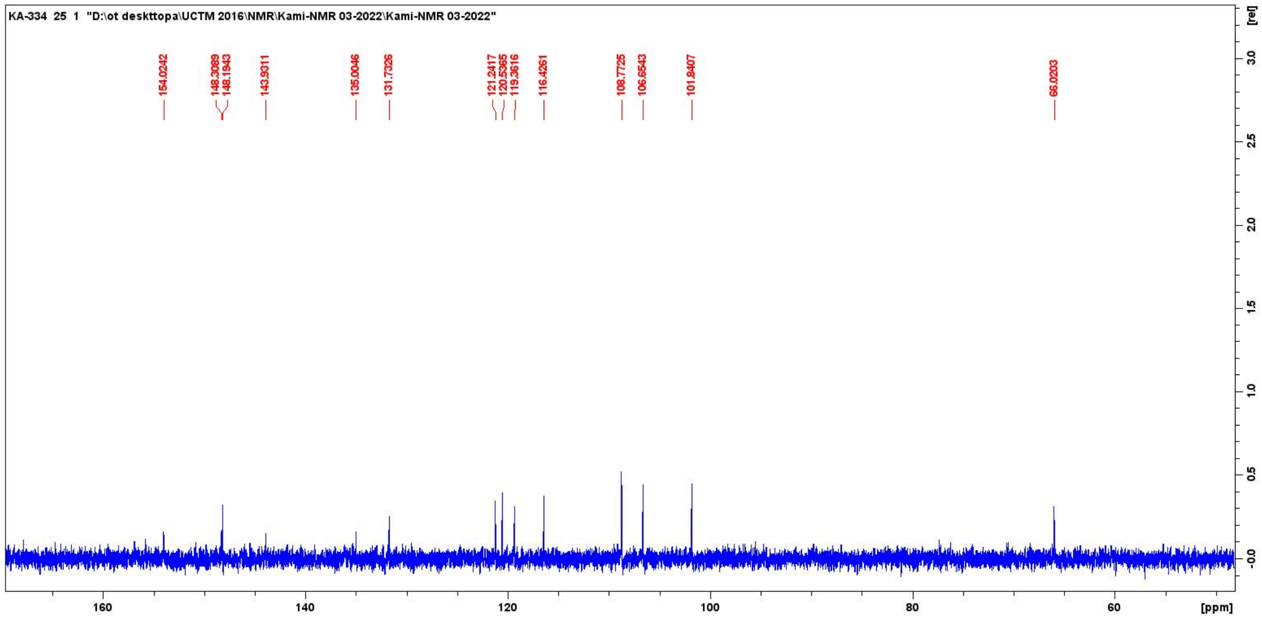


**Figure S18.** $^{13}C$ NMR spectrum of compound **3d.**

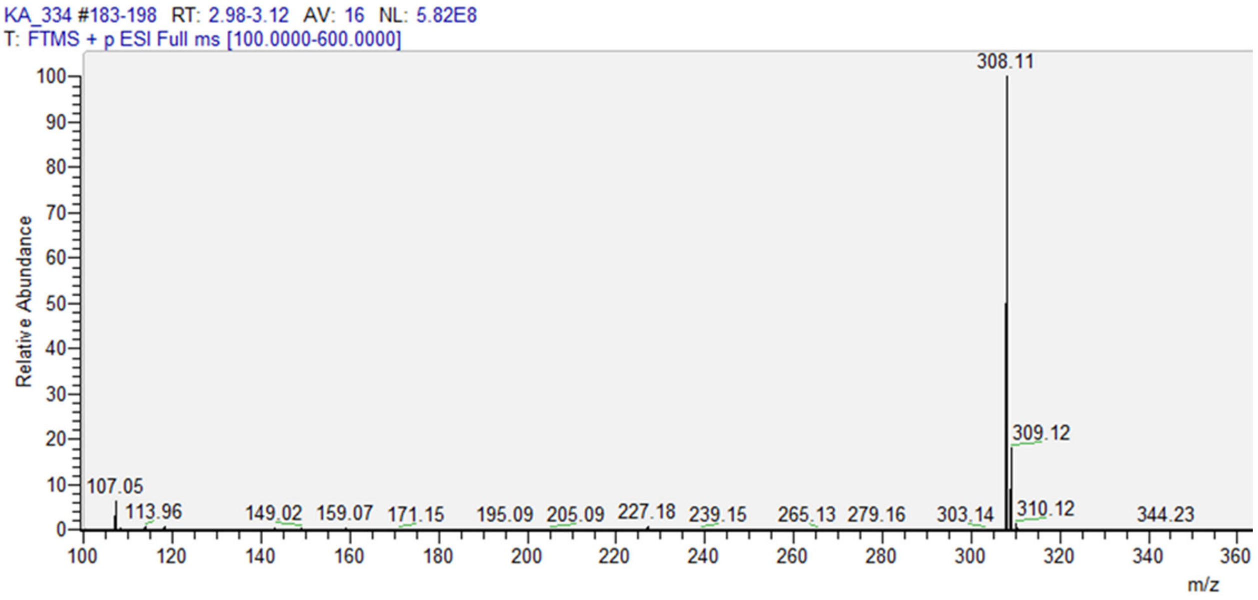


**Figure S19.** HRMS (ESI) spectrum of compound **3d.**

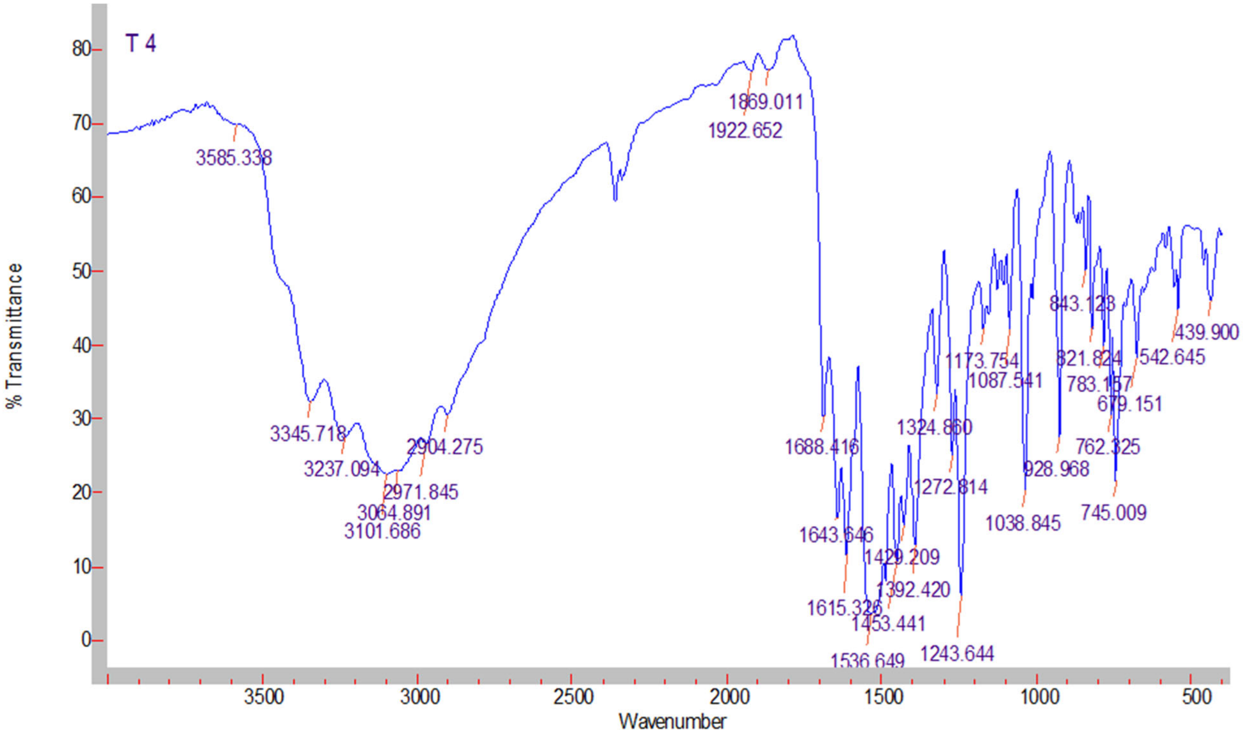


**Figure S20a.** IR spectrum of compound **3d.**

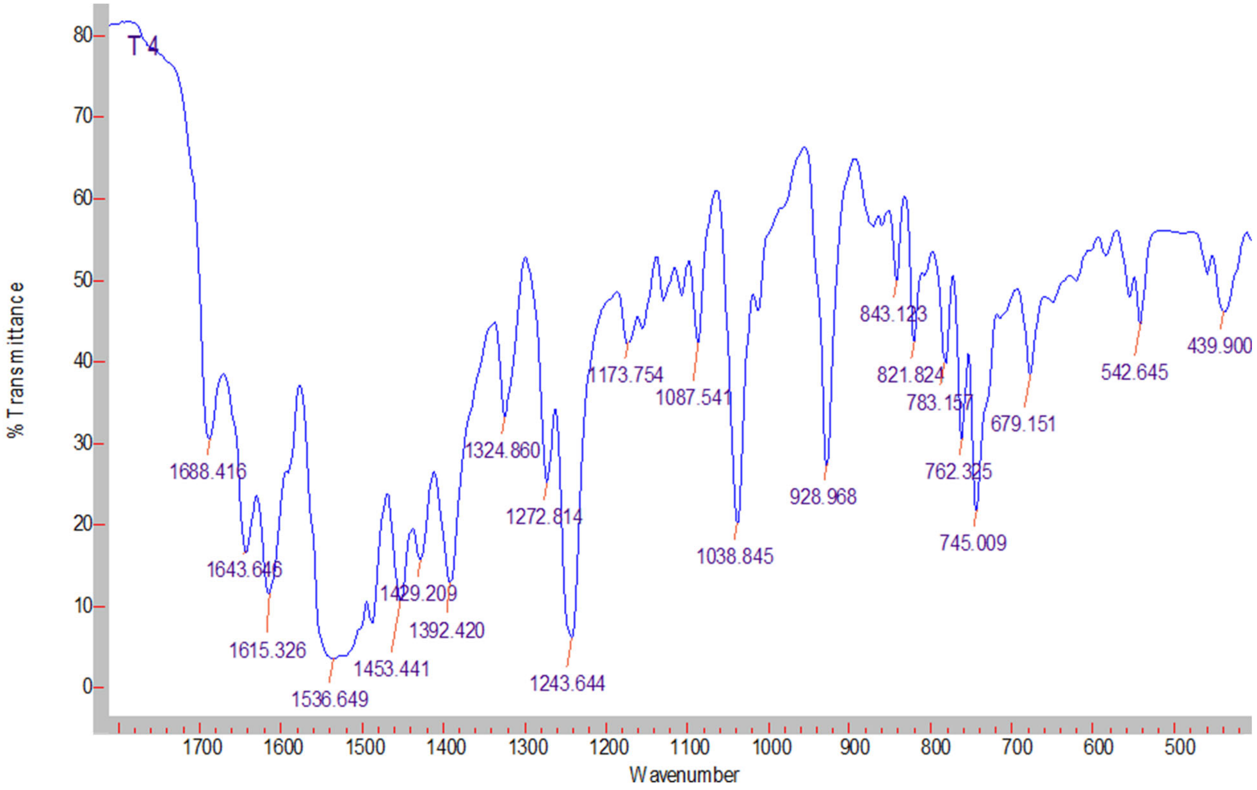


**Figure S20b.** IR spectrum of compound **3d.**

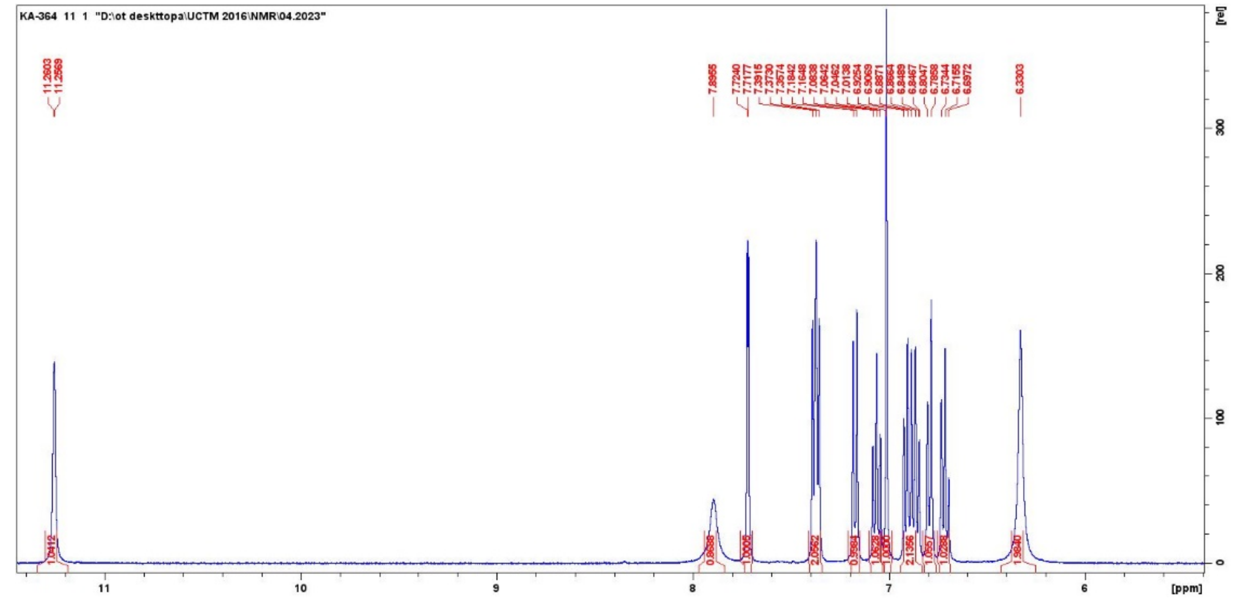


**Figure S21.** $^1$H NMR spectrum of compound **3e.**

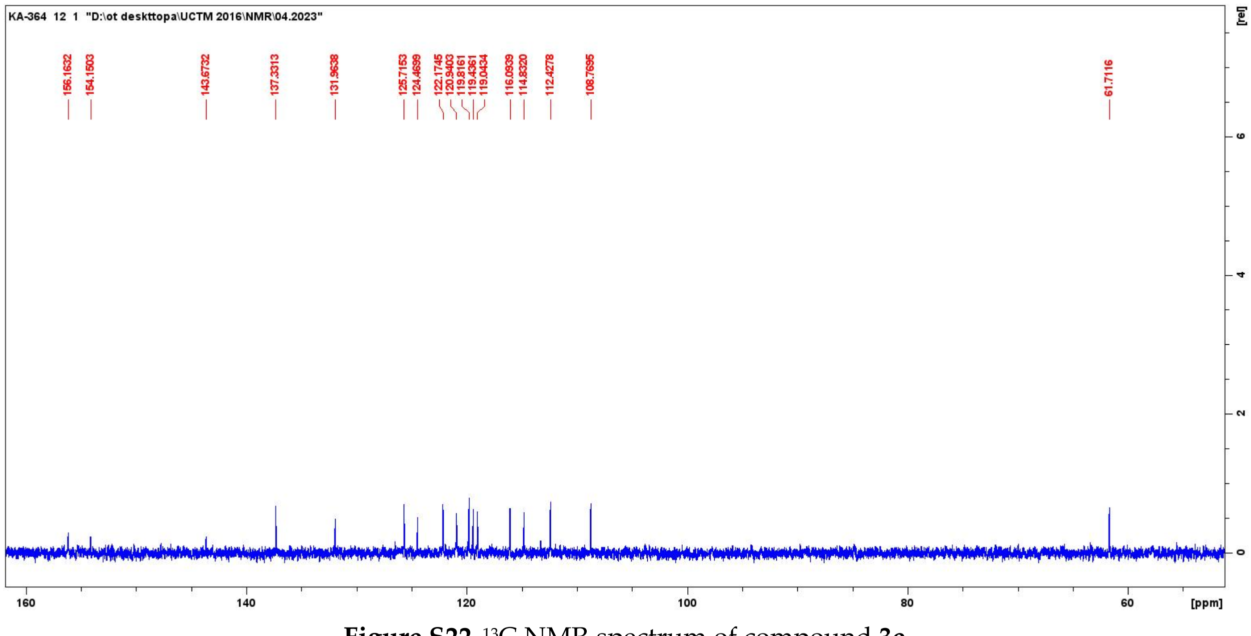


**Figure S22.** $^{13}$C NMR spectrum of compound **3e.**

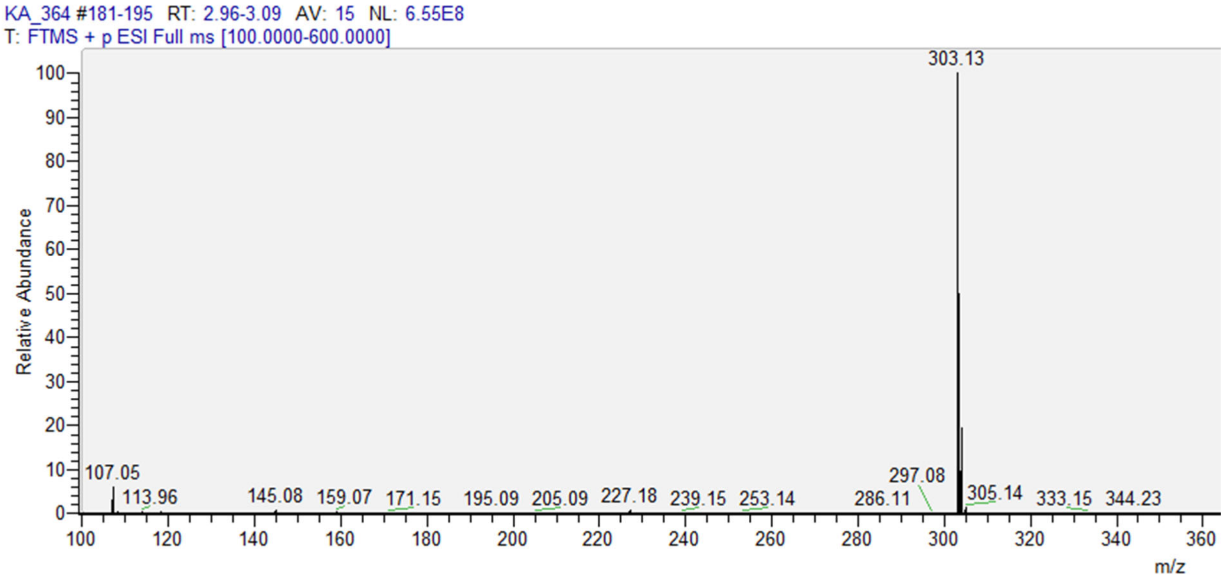


**Figure S23.** HRMS (ESI) spectrum of compound **3e.**

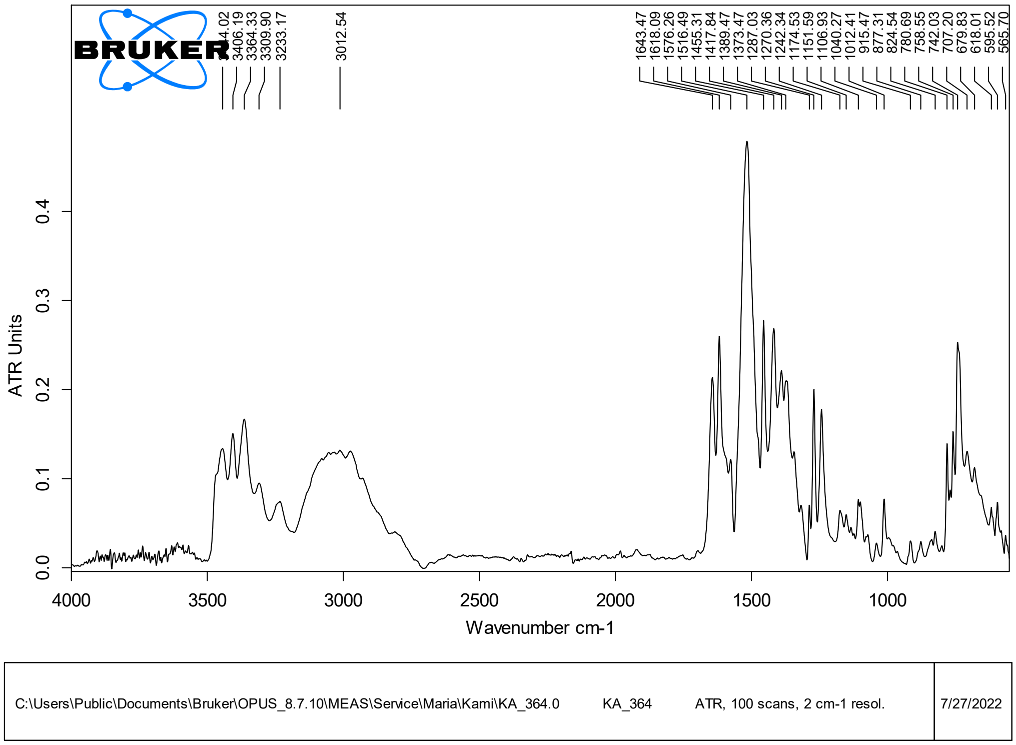


**Figure S24a.** IR spectrum of compound **3e.**

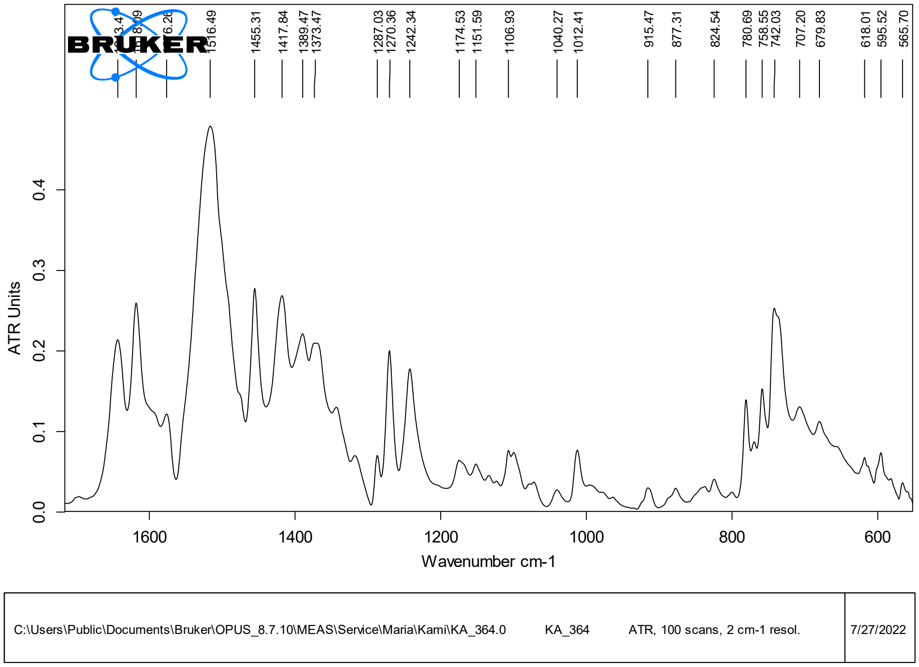


**Figure S24b.** IR spectrum of compound **3e.**

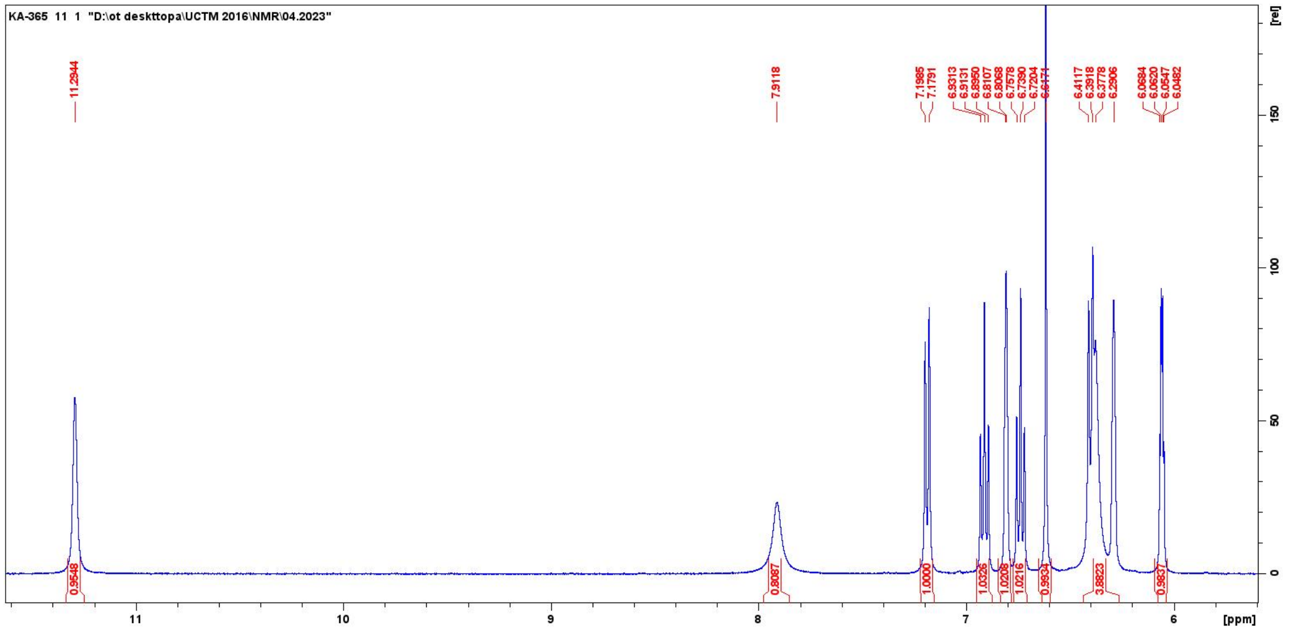

**Figure S25.** $^{1}H$ NMR spectrum of compound **3f.**

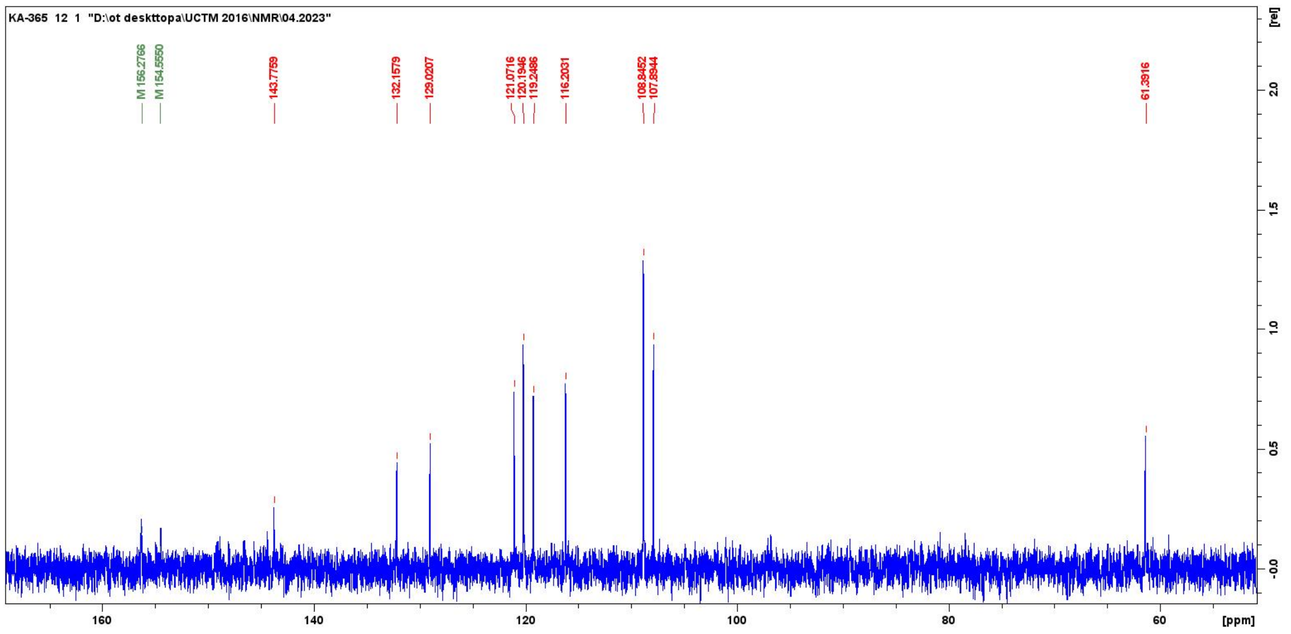

**Figure S26.** $^{13}C$ NMR spectrum of compound **3f.**

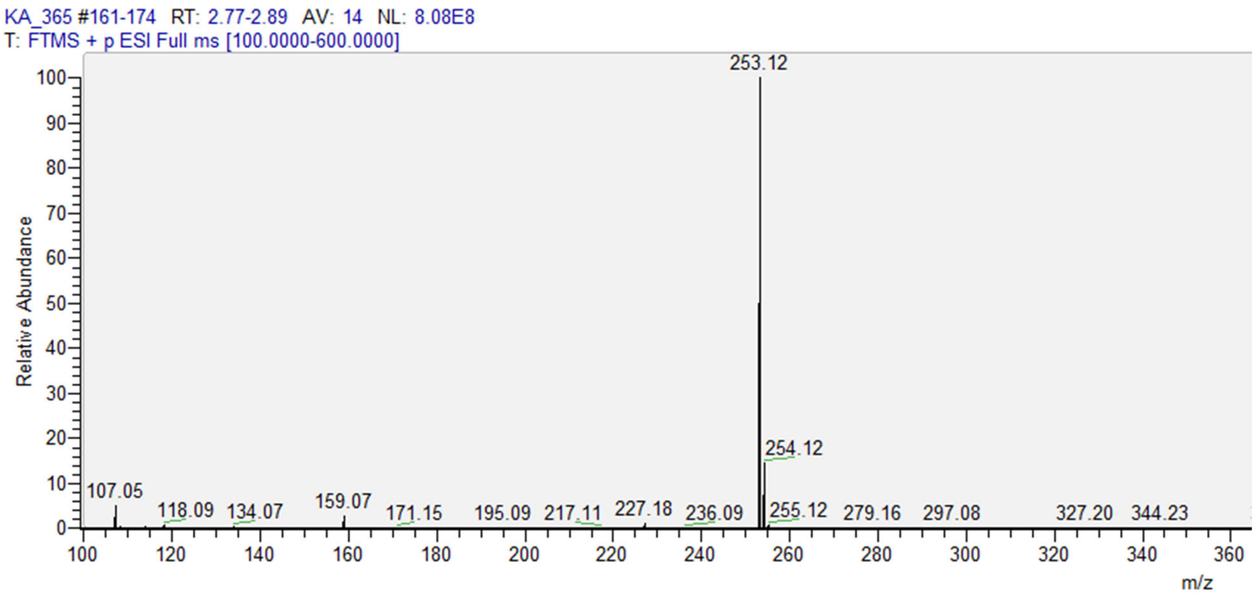


**Figure S27.** HRMS (ESI) spectrum of compound **3f.**

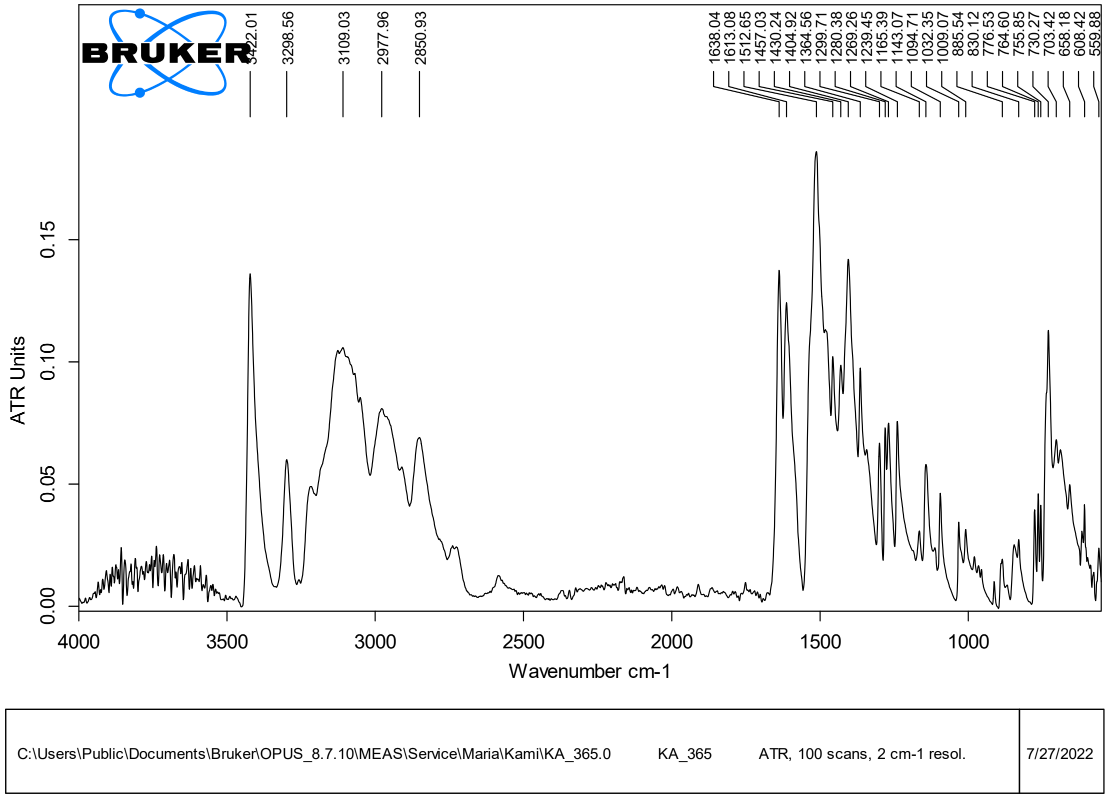


**Figure S28a.** IR spectrum of compound **3f**.

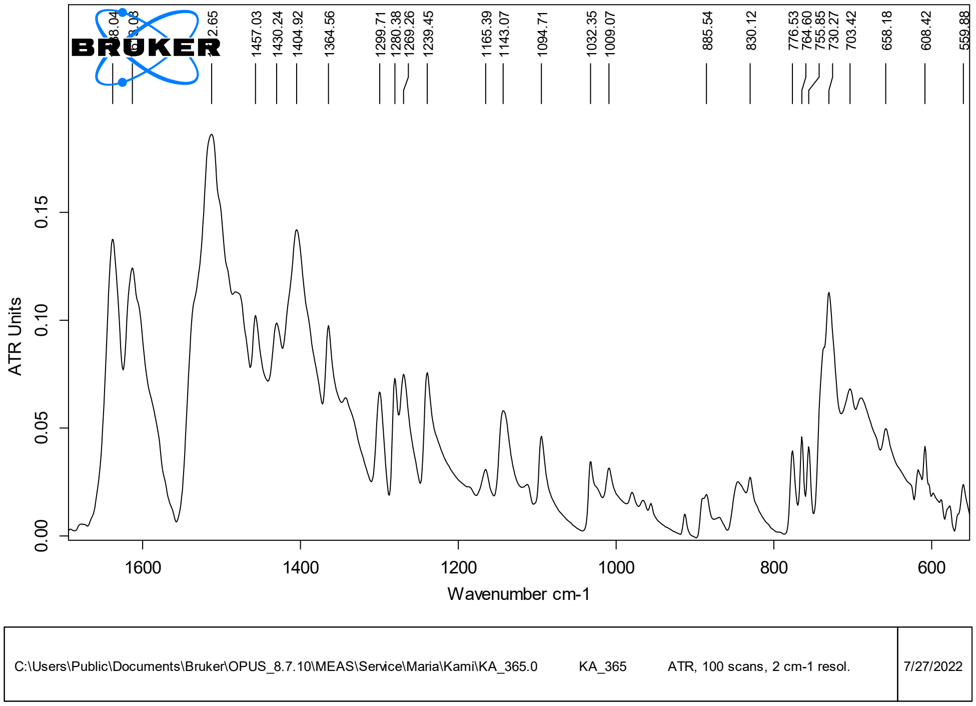


**Figure S28b.** IR spectrum of compound **3f.**
